# The scope of emergency nursing viewed through the lens of complex adaptive systems: A discussion paper

**DOI:** 10.1016/j.ijnsa.2024.100270

**Published:** 2024-11-26

**Authors:** Katarina E Göransson, Jonathan Drennan, Hanne Mainz, Nanna Fauerholdt Skov, Maria Amritzer, Lena M Berg, Karen V Andersen, Marianne Lisby

**Affiliations:** a School of Health and Welfare, Department of Caring Sciences, Dalarna University, 791 88 Falun, Sweden; b School of Nursing, Midwifery and Health Systems, University College Dublin, Dublin, Ireland; c Research Center for Emergency Medicine, Department of Clinical Medicine, Aarhus University, Denmark; d Clinical Nursing Research Unit, Aalborg University Hospital, Denmark; e Emergency Department, Aarhus University Hospital, Denmark; f Karolinska Institutet, Department of Medicine Solna, Stockholm, Sweden; g Karolinska University Hospital, OO H, Emergency and Reparative Medicine Theme Huddinge, Stockholm, Sweden

**Keywords:** Emergency departments, Emergency nursing, Emergency service hospital, Nurse's role

## Abstract

Across the world, emergency department nurses care for patients around the clock all year long. They perform tasks ranging from direct nursing care to managing patient flow, working in an environment characterised by interdependencies among numerous actors. The complex context in which emergency nurses operate has not been thoroughly described or discussed, indicating a knowledge gap. Hence, the aim of this discussion paper is to describe and discuss nursing in the emergency department and the connection between patient flow and nursing care, drawing on the concept of complex adaptive systems.

The acts of direct patient care and patient flow, when viewed through the lens of complex adaptive systems, are central components of emergency nursing. Through a stepwise description of these two perspectives, based on literature and clinical experience from European countries, the paper illustrates the complexity of the emergency nursing context in a novel manner. We argue that direct patient care and patient flow, combined as patient flow management, constitute essential parts of the core of emergency department nursing. Further studies are needed to challenge or confirm this assertion.

**What is already known**

Emergency departments are complex, and factors like chief complaints and the number of patients vary on a daily basis.

Emergency nurses carry out direct patient care and patient flow-related tasks.

Alt-text: Unlabelled box

**What this paper adds**

A state-of-the-art description of nursing in the emergency department.

An illustration of emergency nursing through the lens of complex adaptive systems.

Alt-text: Unlabelled box

## 1 Introduction

Ensuring patient safety and surveillance is essential for nurses in emergency departments, particularly with regard to the correlation between patient flow and direct nursing care. Emergency nursing has been defined in many ways but is generally considered the ‘*care of individuals of all ages with perceived or actual physical or emotional alterations of health that are undiagnosed or that require further interventions*’ (Alpi, 2006). Despite the fact that patient case-mix and annual patient visits per year are rather stable over time in the emergency departments, emergency nurses face challenges in managing the complexity of the emergency department environment.

However, there is a lack of research describing and discussing the complex work of nurses in emergency department settings. To understand the demands of emergency department nursing, it is necessary to understand the challenges inherent to the emergency department context itself. Emergency departments are highly complex, meaning that it is not possible to understand different components separately. Instead, these components must be understood in relation to how they interact with each other, and the more intertwined they become, the more complex and difficult they become to analyse and predict (Sturmberg et al., 2014). A system operating in a complex context, such as the emergency department, is typically referred to as a complex adaptive system (Braithwaite et al., 2017). Therefore, inspired by the work by Albsoul et al. (2021), the aim of this paper is to describe the current state of the art of knowledge in emergency department nursing and discuss the connection between patient flow and nursing care based on the notion of complex adaptive systems.

### 1.1 Complex adaptive systems – theoretical framework

A complex adaptive system is a dynamic system in which individuals and teams within the system perceive, act, react, communicate, adapt, learn and self-organise over time (Braithwaite et al., 2017). Within the health care context, complex adaptive systems have been described as ‘*a collection of individual components with freedom to act in ways that are not always totally predictable, and whose actions are interconnected so that one component's actions change the context for other components*’ (Plsek and Greenhalgh, 2001, p. 625). Thus, a complex adaptive system must be considered in relation to its context, as it consists of several subsystems related to other systems. The behaviour of the system is also influenced by signals from external systems that affect the internal environment. This can be described as an intricate network of health care providers, patients, policies and technologies that interact and adapt, often leading to unpredictable outcomes (Carayon et al., 2006). According to the notion of complex adaptive systems, health care in general, and emergency departments in particular, do not consist of linear processes, and changes in one area can have cascading effects throughout the system.

Resilience, both from the organisation and its members, is needed to successfully deal with a complex system – not least to ensure patient safety. Resilience can be defined as a system's ability to adjust its functioning prior to, during and following disturbances in the system and maintain high-quality performance during both expected and unexpected events (Hollnagel et al., 2006). This ability can be likened to a foam ball that can be squeezed together but expands back to its original shape when the pressure is released.

However, resilience is not constant, as it is created within the system and depends on the existing variation in the context, e.g., the emergency department. Thus, it is not possible to completely compare one emergency department or complex adaptive system with another, even if they share many similarities, as they are part of different systems. Examples of components that contribute to the complexity of the emergency department context include loosely assembled teams with uneven skills and an uneven influx of patients with a wide variety of presentations and severities. These components must also be considered in relation to the fact that emergency departments rely heavily on other surrounding systems, such as radiology and laboratory services and access to inpatient beds, which the emergency department has no control over. The use of the notion of complex adaptive systems in this paper makes it possible to challenge current descriptions of emergency department nursing and provide a state-of-the-art description of emergency department nursing.

### 1.2 The emergency department within the health care system

The organisation of emergency departments varies across countries. One way of defining them is as ‘consultant-led 24-h services with full resuscitation facilities and designated accommodation for Accident and Emergency patients’ (NHS, 2019). In the last decade, a number of emergency departments have also incorporated short stay units, observational or acute admission units in which patients can be observed and cared for over a period of 24–48 hours. Similarly, patient access to emergency departments varies across countries. In some Scandinavian countries, for example, Denmark, patients have to be referred to the emergency department by either their general practitioner, out-of-hours physicians or the emergency medical services (Vork et al., 2011). However, in most countries, patients can generally access emergency departments by self-referral, which makes the intake and flow of patients in emergency departments far more unpredictable and susceptible to crowding. Once patients enter an emergency department, the workflow can be divided into three generic phases: input, throughput and output (Asplin et al., 2003).

In brief, the input phase is defined as activities that occur before the patient arrives at the emergency department, e.g., self-referral or arriving following a general practitioners visit. The throughput phase is the period from the point of arrival at the emergency department and includes triage, diagnostic evaluation and care and treatment activities carried out in the department. Output is defined as activities related to the patient leaving the emergency department to be either admitted, discharged or transferred to another health care setting (Asaro et al., 2007; Asplin et al., 2003). The activities in the throughput phase, particularly the link between patient flow and nursing care and the challenges that nurses face when trying to adapt to the provision of care in a complex environment, are the focus of this paper.

### 1.3 The context of emergency department nursing

Emergency departments are complex and high-intensity settings that increase the risk of adverse events due to the treatment of a large volume of patients simultaneously (Af Ugglas et al., 2020; Morley et al., 2018; Wolf et al., 2017). In addition, health care professionals working in these complex settings are at risk of cognitive overload, time constraints, constant interruptions and uncertainties, with, at times, little knowledge of the patient's past medical history (Croskerry et al., 2008; Nielsen et al., 2013). Within this complex environment, the current and projected future shortage of nurses working in emergency departments increases the likelihood of adverse events and negatively impacts the patient experience (Johnson et al., 2021). Crowding (Berg et al., 2019), extended time to triage, interruptions during triage (Johnson et al., 2021), increasing lengths of stay and boarding patients waiting for a bed (do Nascimento Rocha et al., 2021; Laam et al., 2021; Pryce et al., 2021) are all associated with reduced patient safety. The levels of nurse staffing are associated with patients leaving without being seen, patient care time in the department and patient satisfaction in emergency departments (Drennan et al., 2024; Recio-Saucedo et al., 2015). While patients are waiting for an initial assessment by the primary care provider (i.e. a physician or advanced nurse practitioner in the emergency department), which can be many hours, and also during boarding in the emergency department, registered nurses have the overall responsibility for patient surveillance and the initiation of care processes. Unlike ward settings, where there are generally a fixed number of patients, admissions to emergency department are unpredictable, leading to periodically increased nursing workloads. Recent research on missed nursing care in emergency departments has emphasised that patients’ fundamental needs, assessments and follow-ups often were missed due to the patient load and the design of emergency departments (Amritzer et al., 2024; Duhalde et al., 2023). Thus, ensuring patient safety and surveillance are key parts of the role of emergency department nurses, requiring resilient performance according to the complex adaptive system perspective.

### 1.4 Emergency department nursing

Being a nurse in a complex emergency department environment is a demanding and often unpredictable job requiring knowledge of other acute and community specialty areas (surgical, medical settings, intensive care, primary health care). Moreover, nurses in emergency departments must be capable of working under pressure, communicating effectively with a broad variety of patients, collaborating with many health care providers (Thøgersen et al., 2023) and prioritising the care that patients need according to the severity of their condition (Berg et al., 2016; Berg et al., 2016; Berg et al., 2013; Johnston et al., 2016). Finally, and perhaps most importantly, emergency department nurses must perform accurate and timely assessment of patients to avoid clinical deterioration (Hiraid Research Group, 2021). Concurrently, they must adapt to the heterogeneity of patients who are seeking care and treatment for various symptoms of injury or disease. In addition, the patient population using emergency services is characterised by a wide range of ages, presentations and fluctuations in clinical condition and urgency (Berg et al., 2019; Sorensen et al., 2021). All of the above must be carried out while the number of patients each nurse must care for varies from hour to hour and throughout the day (Amritzer et al., 2021).

As mentioned above, the throughput phase consists of intake, triage, diagnostic evaluation, care and treatment, and for some patients a boarding period as well, while they await admission to a hospital ward. In emergency department nursing, this phase involves triage upon arrival to the emergency department in order to determine the patient's level of urgency, that is, the need to be seen by a physician or an advanced nurse practitioner (FitzGerald et al., 2010). This is followed by a nursing assessment process, as outlined in the HIRAID framework: history (presenting problem as well as individual health history); identifying red flags (indicators of urgency: physiological and historical); assessment (clinical examination, including vital signs); interventions (deliver patient care and treatment based on assessment findings, which may be nurse initiated or by request from a physician); and diagnostics (ordering, performing and reviewing diagnostic tests in a timely manner) (Munroe et al., 2015). For patients waiting to be admitted to an in-hospital department (often known as boarding), their need shifts towards ward-based nursing care (e.g. administer regular medication, reassessments, performing rounds and addressing basic nursing needs). For emergency department nurses, it can be challenging to meet such needs in an often chaotic and unpredictable emergency department environment, primarily driven by patient flow (Eriksson et al., 2018). Moreover, the sudden onset of an illness can be especially challenging for relatives, and thus emergency department nurses may find themselves dealing with relatives of critically ill patients who require special support, inclusion and information alongside providing care to the patient (Blaabjerg et al., 2024, 2022; Emmammaly et al., 2020).

However, emergency department nursing extends far beyond direct patient care and support of relatives. Nurses support and sustain the delivery and organisation of health services and coordinate the necessary processes to ensure flow in the emergency department, fulfilling an important operational and strategic role (Benjamin and Wolf, 2022). This role related to patient flow, which is often invisible to patients, has become increasingly essential to ensure available beds for incoming patients and to reduce the risk of crowding (Melon et al., 2013; Sharma et al., 2020). Patient flow has been described as the progressive movement of patients through care processes (De Freitas et al., 2018), while patient flow management refers to the application of holistic perspectives, dynamic data and complex consideration of multiple priorities to ensure timely, efficient and high-quality patient care (Benjamin and Jacelon, 2022). Hence, in addition to the complexity emergency department nurses face in providing direct patient care, they must manage patient flow to ensure that patients are receiving appropriate diagnostic care and interventions during their time in the emergency department as well as creating a safe environment as new patients arrive in the department.

### 1.5 Patient flow in the emergency department

The flow process has become even more evident in the emergency department context during the past decade, partly due to quality improvement targets, such as *time to be seen by a physician or advanced nurse practitioner* and *length of stay* (Melon et al., 2013; NHS, 2019). Patient flow occurs during the throughput and output phases described by Asplin et al. (2003). As mentioned above, the triage level refers to the maximum length of time estimated to be medically safe for a patient to wait before being seen by the primary care provider. Hence, the triage level relates to patient flow, indicating the amount of time in which the patient is expected to be taken care of during the initial part of the throughput phase. However, the timeframes stipulated within each triage level can be difficult to maintain, especially given crowding and patient complexity, and reassessments of waiting patients are increasingly required to ensure patient safety and to prevent patients from deteriorating. The timeframe for the patient's entire emergency department visit, the emergency department length of stay, is another example of the flow perspective. Since the implementation of length of stay as a quality indicator, there has been an increased focus on the management of patients in the emergency department. The definition of an extended emergency department length of stay varies widely across countries but is often between four and 48 hours (Andersson et al., 2020).

Nurses play an important role in ensuring effective patient flow forward, and emergency department nurses are responsible for the logistics of this process. Benjamin and Wolf (2022) suggests that research on patient flow should also include the perspectives of frontline nurses. Notably, frontline nurses have been found to view themselves as external agents of the flow process (Sharma et al., 2020). Patient flow occurs as time goes by during the patient's visit. Eventually, patients will reach their final emergency department outcome, that is, discharge or admission (including transfer). The patient flow may be fast or slow and may also be predictable or unpredictable for the nurse. Often, factors outside the emergency department nurses’ control influence patient flow (e.g. lack of beds, short-staffing, increased admissions) (Sharma et al., 2020). Hence, nurses must adapt their interventions and other actions in relation to variable patient flow aspects. Patient flow management allows nurses to incorporate patient flow with their other tasks. Benjamin and colleague defined patient flow management as ‘the application of holistic perspectives, dynamic data, and complex considerations of multiple priorities to enable timely, efficient, and high-quality patient care’ (Benjamin and Jacelon, 2022). Further, the role of frontline nurses in patient flow management has been highlighted (Benjamin and Wolf, 2022).

### 1.6 Combining nursing and patient flow in the emergency department

There is a lack of studies on emergency department patient flow in relation to direct nursing care in the emergency department. From an emergency department nursing research perspective, we believe it is important to accurately describe the nurse's role in the emergency department, especially to understand the impact of nursing on patient outcomes (Drennan et al., 2024). Emergency department nurses often highlight the complexity of nursing work in emergency departments, which involves both patient needs and patient flow processes. In addition, they emphasise factors beyond the emergency department, such as waiting times for X-rays, ambulance diversion and a lack of hospital beds due to nursing shortages. Overall, emergency department nurses work in a complex environment in which they have some, but often a subordinate, influence. Given the priority of improving patient flow, that is, shortening the emergency department length of stay, it is important to discuss and gain a better understanding of how emergency department nursing can be further developed.

**Fig. 1:**
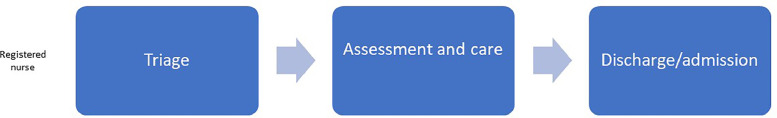
Illustration of a patient flow process during an emergency department visit.

Emergency department nursing involves more than just ensuring patient flow, and the perspective of patient flow should be considered in relation to other aspects of emergency department nursing (Benjamin and Wolf, 2022). This is particularly important for exploring the relationship between emergency department nursing and patient outcomes. Emergency department patient flow is often visualised in a linear way, as illustrated schematically in Fig. 1. For example, a recent study described the patient's process through the emergency department, from triage to the patient leaving the emergency department (Curtis et al., 2023). Two different flows are described, with the novel flow (EPIC-START) visualising the emergency department nurse's role more clearly (Curtis et al., 2023). In addition, EPIC-START illustrates some of the additional tasks emergency department nurses perform in the emergency department context. The linear description of the flow process indicates the order in which actions are taken and the dependencies between actions.

**Fig. 2:**
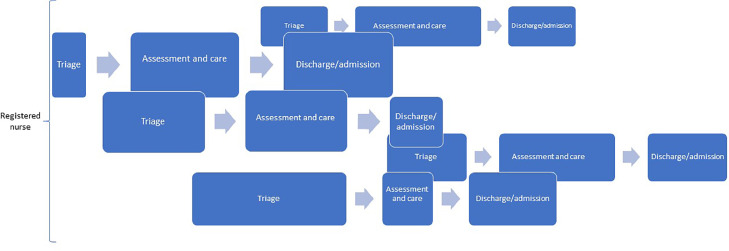
A schematic illustration of multiple emergency department patient flow processes from the perspective of emergency department nurses. The width of the boxes illustrates length of time for each process.

However, a single linear model cannot capture the fact that patients move along the flow process at different speeds or that they enter the process at different times. At times, the flow can even be circular rather than linear. This means that an emergency department nurse may have to navigate several patient flow processes simultaneously (Benjamin and Jacelon, 2022) (Fig. 2).

**Fig. 3:**
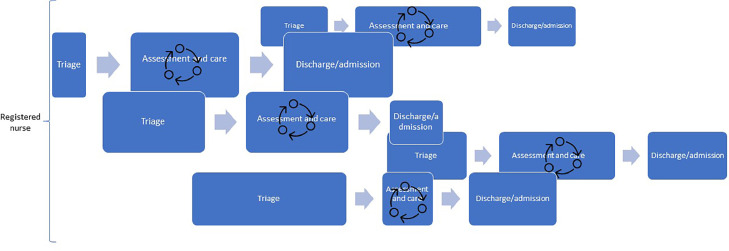
A schematic illustration of the back-and forth movement within each patient's emergency department flow.

In Scandinavia, for the past 15 years, many emergency departments have followed the EPIC-START model. This has mainly been due to the implementation of process-based triage systems, such as Adaptive Process Triage, the Rapid Emergency Triage and Treatment System and Danish Emergency Process Triage (Engebretsen et al., 2013; Plesner et al., 2015; Wireklint et al., 2021). Based on our clinical experiences, we have noticed that emergency department care, including both emergency nursing and emergency medicine, is seldom undertaken in a linear way. Fig. 3 illustrates this complexity in patient flow, where one emergency department nurse is responsible for a number of patients (Amritzer et al., 2021; Berg et al., 2019). Hence, the notion of linearity within the emergency department does not address the complexity of actual patient flow and its impact on emergency department nursing. In reality, emergency department nurses handle multiple patients who are in different phases of the flow process (Benjamin and Jacelon, 2022). Within one patient's flow process, there may be a back-and-forth movement, illustrated by the circle of arrows in Fig. 3. For example, a patient might undergo a chest X-ray as part of their assessment, which will influence the patient's emergency department length of stay (Pryce et al., 2021). While waiting for the x-ray result, the patient may undergo one or several interventions (e.g. pain medication, vital sign reassessment, re-positioning and oxygen therapy) (Duhalde et al., 2023). The result of the X-ray may require further tests or interventions. In addition, the amount of time each patient spends in the different phases may differ (e.g. a patient with a rapid triage phase may experience long assessment and care phases). If the patient spends many hours in the emergency department, which is not uncommon due to crowding, both basic (e.g. mouth care and pressure ulcer prevention) and specific nursing (e.g. patient or family education and discharge planning) interventions may be required. The execution of these nursing interventions is dependent on both the patient's care needs and flow process.

**Fig. 4:**
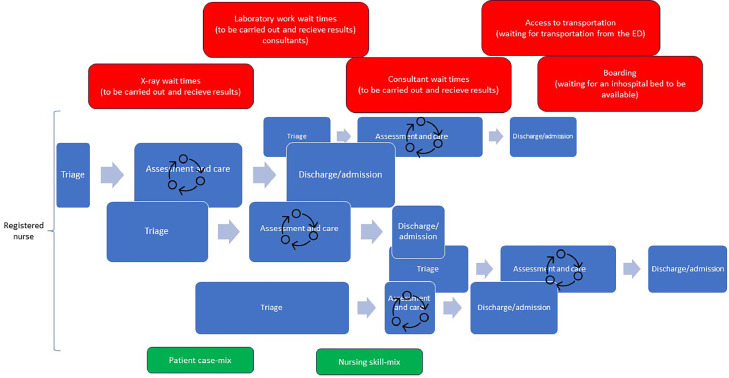
Illustration of external factors that influence the patient flow process in emergency departments.

Further adding complexity to emergency department nursing, the non-linear flow process is often influenced by many factors, some of which are outside the control of the staff in the emergency department (Benjamin and Jacelon, 2022). Fig. 4 illustrates the emergency department from a complex adaptive system perspective. The red boxes represent external factors that influence the patient flow process. There may also be internal factors, such as a large number of high-severity patients and a wide patient case mix as well as varying staff availability, including skill mix (green boxes). Emergency department nurses must balance a wide range of tasks and adjust them based on several factors, some of which some are outside of their control. Hence, nurses balance the tasks they have control over, resulting in direct patient care often being delayed or missed (Duhalde et al., 2023).

Fig. 1, Fig. 2, Fig. 3, Fig. 4 illustrate patient flow from the perspective of front line emergency department nurses. By adding components one by one, a fuller picture of emergency department nursing as a complex system emerges. As depicted, patient flow and frontline nursing tasks are intertwined, often more or less simultaneously. Every patient has a specific pace and time stamp during the emergency department process, and the emergency department nurse handles multiple patient flows simultaneously during a shift. By applying the complex adaptive systems perspective to emergency department nursing, it becomes clear that the concepts of patient flow and frontline nursing must be considered to capture the work carried out by emergency department nurses. Further, external factors also influence patient flow management in the emergency department. Hence, even if factors like case-mix and number of patients visits per year may be stable over time, other factors contribute to unpredictability and complexity. Overall, our paper shows that emergency department nurses work in a complex system that requires them to be adaptable. It also adds to the body of knowledge on emergency department nursing, providing a state-of-the-art description of the environment in which emergency department nursing is practiced. The difficulties experienced when carrying out research about emergency department processes or applying workflow models in the emergency department, may be explained by the complexity and non-linearity presented in this paper. Knowing and understanding the environment are important from a research perspective, particularly in studies investigating the association between emergency department nursing and patient outcomes (Fig. 4).

## 2 Summary and conclusion

In summary, this paper has described the complexity of emergency department nursing and the connection between patient flow and direct nursing care based on the authors’ clinical experience as emergency department nurses from different European settings as well as relevant research literature. This state-of-the-art description of emergency department nursing was achieved by applying the complex adaptive systems perspective, and we argued that direct patient care and patient flow, combined as patient flow management, are essential aspects of emergency department nursing. Further studies are needed to challenge or confirm this assertion.

## 3 Implications for future research

Research on the impact of emergency department nursing on patient outcomes has increased in recent years but remains scarce. Future studies measuring the impact of nursing care in the emergency department should include the patient flow perspective in addition to direct patient care taking the complexity of nursing in these departments into account. Finally, it is also important to explore the core aspects of emergency department nursing from the perspectives of the receiver (patients) of emergency department nursing services, which was beyond the scope of this paper.

## CRediT authorship contribution statement

**Katarina E Göransson:** Writing – review & editing, Writing – original draft, Visualization, Validation, Project administration, Methodology, Formal analysis, Data curation, Conceptualization. **Jonathan Drennan:** Writing – review & editing, Writing – original draft, Validation, Formal analysis, Conceptualization. **Hanne Mainz:** Writing – review & editing, Writing – original draft. **Nanna Fauerholdt Skov:** Writing – review & editing, Formal analysis, Conceptualization. **Maria Amritzer:** Writing – review & editing, Writing – original draft, Formal analysis, Conceptualization. **Lena M Berg:** Writing – review & editing, Writing – original draft, Formal analysis, Conceptualization. **Karen V Andersen:** Writing – review & editing, Writing – original draft, Conceptualization. **Marianne Lisby:** Writing – review & editing, Writing – original draft, Validation, Project administration, Formal analysis, Data curation, Conceptualization.

## Declaration of competing interest

The authors declare the following financial interests/personal relationships which may be considered as potential competing interests:

Jonathan Drennan reports financial support was provided by Health Research Board, Ireland. Jonathan Drennan reports financial support was provided by Department of Health, Ireland. If there are other authors, they declare that they have no known competing financial interests or personal relationships that could have appeared to influence the work reported in this paper.
